# 羊水栓塞导致弥散性血管内凝血1例报告并文献复习

**DOI:** 10.3760/cma.j.cn121090-20241130-00501

**Published:** 2024-12

**Authors:** 淑芳 李, 星 辛, 娟 肖, 文成 丁, 剑利 乌, 少帅 王, 素华 陈, 玲 冯, 星光 林

**Affiliations:** 华中科技大学同济医学院附属同济医院妇产科，武汉 430000 Department of Obstetrics and Gynecology, Tongji Hospital of Tongji Medical College, Huazhong University of Science and Technology, Wuhan 430000, China

## Abstract

羊水栓塞是一种罕见但极其凶险的产科灾难性疾病，凝血功能障碍是其常见临床表现。本文报告1例以弥散性血管内凝血为首发表现的羊水栓塞病例，并进行文献复习，以期提高临床医师对产科凝血功能障碍的关注，为羊水栓塞的早期识别和处理提供依据。

## Abstract (en)

Amniotic fluid embolism（AFE）is a rare but extremely dangerous obstetric catastrophic disease, with coagulation dysfunction being a common clinical manifestation. This article reports a case of AFE with disseminated intravascular coagulation as the initial presentation, and conducts literature review. In order to increase the attention of clinical physicians to obstetric coagulation dysfunction and provide a basis for early identification and treatment of AFE.

羊水栓塞（amniotic fluid embolism，AFE）是产科特有的罕见、危险的并发症，发生率为（1.9～7.7）/10万，死亡率为19％～86％[1]，AFE中出现凝血功能障碍者高达83％，且可为首发表现[2]–[3]。AFE发生后，羊水成分使凝血系统激活、血小板活化、纤维蛋白沉积，导致DIC发生，凝血因子和血小板消耗、纤溶系统激活导致纤溶亢进，造成难以控制的出血、休克、微血管栓塞和微血管病性溶血。早期发现DIC并进行有效的救治非常重要。

本文报道1例以DIC为首发表现的AFE病例，讨论临床救治过程中的注意事项和改进方向。

## 病例资料

患者，女，41岁，主因“停经39周，阵发性腹痛2 h”入院。患者自然受孕，早孕期经超声核对孕周准确，孕期规律产检，孕期NT 1.1 mm，因高龄于孕18周行羊水穿刺查染色体核型和拷贝数变异（CNV）未见明显异常，孕23周行胎儿系统超声未见明显异常，孕24周行口服葡萄糖耐量试验（OGTT）检查结果正常。因“孕39周，阵发性腹痛2 h”于2020年3月10日在我院急诊就诊，查宫口开2 cm入院。患者既往否认特殊病史，G3P2，经阴道分娩2次。入院后完善相关实验室检查均未见明显异常，其中HGB 120 g/L，PLT 328×10^9^/L，纤维蛋白原（FIB）4.84 g/L，D-二聚体（D-Dimer）1.47 µg/ml，羊水指数20 cm。监测产程进展，第一产程时长4 h 50 min，第二产程时长30 min，分娩一活婴，新生儿无窒息，出生体重3 600 g，会阴Ⅰ度裂伤。

胎儿娩出后4 min，胎盘部分剥离，阴道出血400 ml，可见血凝块。启动产后出血一级预警，予心电监护，心率94次/min，血压120/72 mmHg（1 mmHg=0.133 kPa），经皮血氧饱和度99％，增加1条静脉通道，急查血常规、凝血功能并备血，寻找出血原因。检查发现子宫上段收缩好，但仍有暗红色的血液缓慢流出，考虑子宫下段收缩欠佳，予卡前列素氨丁三醇注射液250 µg肌肉注射，胎盘未完全剥离，行手剥胎盘，胎盘胎膜娩出完整，宫颈无裂伤，会阴Ⅰ度裂伤，裂伤处无明显活动性出血。

胎儿娩出后8 min阴道共出血600 ml，启动产后出血二级预警。患者神智清楚，心率上升到110次/min，血压下降至99/59 mmHg，经皮血氧饱和度98％。继续寻找出血原因，并进行抗休克治疗，补充晶体液1 000 ml，考虑出血原因主要是子宫下段收缩不佳，予卡贝缩宫素注射液静脉滴注促进子宫收缩，同时持续双手按摩子宫。观察10 min再次出血200 ml，出血特点为持续少量的暗红色血液，无明显凝血块。此时患者心率进一步升高至120次/min，血压90/55 mmHg，考虑子宫下段收缩差、药物治疗效果不佳，拟行球囊压迫子宫下段止血。

上球囊压迫止血的过程中，胎儿娩出后25 min，患者突发呛咳，经皮血氧饱和度下降至85％，再迅速恢复到97％，考虑AFE。紧急予地塞米松20 mg静推，患者出现意识淡漠，心率升高至140次/min，血压测不出，予去甲肾上腺素10 µg/min静脉泵入，静脉输血，同时紧急转运至手术室。

胎儿娩出后30 min，出血400 ml时血常规结果示HGB 110 g/L，PLT 157×10^9^/L。胎儿娩出后35 min，患者被送达手术室，意识淡漠，心率160次/min，血压45/20 mmHg，经皮血氧饱和度88％，阴道持续活动性出血，无凝血块，子宫收缩差。血气分析示HGB 81 g/L，pH=7.16，予气管插管、抗休克（2条静脉通道快速输血输液，去甲肾上腺素联合多巴酚丁胺升压）、改善心肺功能、纠正凝血功能障碍和酸中毒等治疗，同时产科医师进行手术止血，考虑药物止血无效、出血危及生命，行子宫次全切，于盆腔留置乳胶引流管1根。术中共出血2 000 ml，输注晶体液1 500 ml，胶体液1 000 ml，悬浮红细胞18 U，新鲜冰冻血浆1 400 ml和纤维蛋白原2 g，尿量800 ml。胎儿娩出后40 min，出血400 ml时的凝血功能示，PT 28.5 s，APTT 58.6 s，FIB<0.50 g/L，D-Dimer>21.0 µg/ml，纤维蛋白（原）降解产物（FDP）>150.0 µg/ml，证明患者在出血400 ml时或在此之前已经出现DIC、纤溶亢进。

手术结束后复查HGB 100 g/L，PLT 58×10^9^/L，PT 17.1 s，APTT 51.2 s，FIB 1.42 g/L，D-Dimer>21.0 µg/ml，FDP>150.0 µg/ml，高敏肌钙蛋白I（hsTnI）356.7 ng/L，肌酸激酶同工酶（CK-MB）11.3 ng/ml，床边心脏超声提示射血分数（EF）56％，肺动脉压正常。后予抗感染、对症支持等治疗，于术后7 d好转出院。出院诊断为：①AFE；②DIC；③产后出血；④孕39周已娩，G3P3，LOA，一活婴。

## 讨论及文献复习

纵观此患者的诊疗过程，AFE诊断明确，救治比较及时，成功挽救了患者的生命。但我们依旧可以从中吸取经验进一步提高临床救治水平。

诊断AFE需要满足以下5条：①急性发生的低血压或心脏骤停；②急性低氧血症：呼吸困难、紫绀或呼吸停止；③凝血功能障碍：有血管内凝血因子消耗或纤溶亢进的实验室证据，或临床上表现为严重出血，但无其他可解释的原因；④上述症状发生在分娩、剖宫产术、刮宫术或产后短时间内；⑤对于上述出现的症状和体征不能用其他疾病解释[1]。该患者在分娩后出血400 ml时所查血常规和凝血功能提示已经出现DIC，紧接着出现低血压、低氧血症，既往无凝血功能障碍病史，根据后续诊疗可以除外心脑血管意外、主动脉夹层、恶性心律失常、肺栓塞、过敏反应等其他病因，故AFE诊断明确。

该患者诊断AFE是在产后近25 min，患者呛咳后发生低血压、低氧血症后才考虑到，在此之前患者主要表现为阴道出血。在出血400～800 ml的16 min内，患者的心率逐渐升高，血压较基准水平下降20 mmHg。单纯的产后出血，基础HGB为120 g/L，逐渐出血至800 ml一般不会引起明显的心率、血压改变，产后出血休克代偿期的病理生理改变是微循环少灌少流、外周阻力和心肌收缩力增强，导致脉压缩小、脉搏增快、血压正常或略下降或略升高。与此例患者血压、心率的变化有一定差异，可能是提醒医师早期发现问题的一个方面。另外就是出血的性状，该患者在出血400～800 ml的过程中未见明显的凝血块，这一临床表现对于判断凝血功能障碍非常关键。AFE的诊断似乎可以提前，但在临床实践中这一点非常困难，无消耗性凝血功能异常的确切证据，无呼吸循环衰竭的表现，诊断依据不足。

该患者在表现为明显的呼吸循环衰竭后诊断AFE，但经过及时有效的抢救，患者在术后7 d出院，主要得益于诊断后的快速反应：①产房因分娩镇痛有24 h在岗的麻醉医师，在患者呼吸循环衰竭时能及时维持患者的呼吸和供氧；②患者出现循环衰竭时快速补充血容量和泵入升压药物，由专人快速运输血液制品，及时输血，短时间内恢复患者的平均动脉压，维持器官灌注，减少患者组织缺血缺氧和再灌注的损伤；③迅速到达手术室获得更高级的生命支持、有效的液体复苏并纠正凝血功能，果断切除子宫，从源头止血，避免产后出血在AFE基础上造成进一步损伤。

该患者是1例以DIC为首发表现的AFE，在出血400 ml或更早时已经发生，然后表现为低血压、低氧血症和产后出血。此例患者的救治中非常重要的一点是早期发现DIC，但目前DIC的诊断和评分系统均基于已有凝血功能检测结果，如PLT下降、PT延长、FIB降低、FDP和D-Dimer增加等[4]，急性出血往往不适用，需根据临床表现和经验判断。

DIC中血小板减少是最常见的实验室检查指标，但刚发生DIC时PLT不会降得很低，甚至在正常范围内[4]。本例患者在出血400 ml时APTT、PT已经明显延长，FIB检测不出，PLT还在正常范围内，但已经较基础水平下降50％。2023年由中华医学会血液学分会血栓与止血学组组织撰写的《产科弥散性血管内凝血临床诊断与治疗中国专家共识》指出，24 h内PLT下降超过50％也是诊断DIC的重要指标之一。在血常规可以10 min出结果的情况下，关注PLT较基础水平的变化非常重要，而不仅看是否在正常范围内[5]。该患者存在AFE基础疾病，有出血倾向及呼吸循环衰竭临床表现、PLT在24 h内下降≥50％，PT 28.5 s，APTT 58.6 s，FIB<0.50 g/L，D-Dimer>21.0 µg/ml，FDP>150.0 µg/ml，根据产科DIC评分表评分高达12分，DIC诊断明确。此时处于低凝期和纤溶亢进期，需输注血浆、冷沉淀、纤维蛋白原等纠正凝血功能并予抗纤溶治疗。

在产后出血中，氨甲环酸的早期应用被提到了重要位置，2017年发表在Lancet中的一项研究显示，早期应用氨甲环酸可降低产后出血导致孕产妇死亡的概率，且未发生严重的不良事件[6]。基于此项研究结果，我国2023年发表的《产后出血预防与处理指南（2023）》中也推荐早期应用氨甲环酸[7]。但在2020年该药物尚未在国内广泛使用，故该患者在救治中并未应用此药物，但从该患者的化验结果中可看到，纤溶亢进从出血400 ml至手术结束持续存在，氨甲环酸的应用指征明确。

在DIC、产后出血的液体复苏中，临床上红细胞、血浆和血小板的输入比例多为1∶1∶1[8]。该患者分娩后和手术过程中共出血3 000 ml，共输注悬浮红细胞18 U，新鲜冰冻血浆1 400 ml，纤维蛋白原2 g。术后实验室检查示HGB 100 g/L，PLT 58×10^9^/L，PT 17.1 s，APTT 51.2 s，FIB 1.42 g/L，虽未严格按照1∶1∶1的比例输入，但除FIB未达到2 g/L的标准外均达到了液体复苏的目标：外周血HGB 70～80 g/L，PLT>50×10^9^/L，PT、APTT在正常上限1.5倍以内。故1∶1∶1并不一定是最佳输血方案。现已有不少文献报道即时分析的血栓弹力图在产科中的应用，认为即时分析的血栓弹力图可以更快和更全面地获得凝血情况，可用于指导血液制品的补充[9]，并已有其指导AFE凝血功能障碍血液制品输注的报道[10]。也有研究显示，应用血栓弹力图指导产后出血的输血可减少红细胞和血浆的输注，可避免输血相关循环超负荷的发生[11]–[14]。但在产科临床工作中并未得到推广使用，还需要更有说服力的研究和证据，是未来的研究方向之一。
